# Supplementary material for: Integrated Transcriptome and Proteome Analysis Reveals the Regulatory Mechanism of Root Growth by Protein Disulfide Isomerase in Arabidopsis
Source: Int J Mol Sci. 2024 Mar 22;25(7):3596. doi: 10.3390/ijms25073596 (PMC11011405; doi:10.3390/ijms25073596)
Supplement: Supplementary file 1 [file ijms-25-03596-s001.zip › Supplementary data.pdf]

Supplementary data

Table S1. Significantly up- and down-regulated top five DEGs

Gene id	Gene names	BaseMean	FoldChange(FT/CT)	Log2FoldChange	Pval	Padj	Regulated
AT1G68250	hypothetical protein	91.72106	68.16459	6.090951	2.62E-29	1.7E-26	up
AT2G36640	ECP63	64.23809	65.05741	6.023641	1.88E-13	2.24E-11	up
AT2G40220	ABI4	7.29913	58.91087	5.880462	7.64E-09	4.29E-07	up
AT1G54870	CHLADR	25.49204	41.04326	5.359073	9.79E-09	5.45E-07	up
AT1G63600	Receptor-like protein kinase-related family protein;	4.161972	33.86886	5.081887	0.000133	0.002719	up
AT3G03670	PER28	53.03814342	0.004736651	-7.721916877	1.17006E-52	2.48893E-49	down
AT3G48640	transmembrane protein	10.55127276	0.012846597	-6.282469948	1.75431E-12	1.89166E-10	down
AT2G29220	L-TYPE	16.47621944	0.015409593	-6.020027463	2.40366E-18	5.73911E-16	down
AT2G13810	AGD2-LIKE	23.15457497	0.017308133	-5.852406102	3.88752E-08	1.95622E-06	down
AT1G21240	encodes a wall-associated kinase	7.636733122	0.01783692	-5.80898968	5.4117E-09	3.14214E-07	down

Table S2. Significantly up- and down-regulated top five DEPs

Accession	Protein names	FC	Log2FC	fdr	Regulated
Q8VYS0	Probable cysteine protease RD19D	2.488019	1.314997	0.001503	up
Q9LY27	NFU1 iron-sulfur cluster protein	2.486637	1.314196	0.001757	up
Q9SUI4	PSI-L	2.428813	1.280251	0.153237	up
F4JQF1	Late embryogenesis abundant (LEA) protein	2.336686	1.224464	0.003464	up
A0A178W2W5	Ferredoxin	2.162579	1.112753	0.085177	up
A0A1P8AU94	Kinase family with ARM repeat domain-containing protein	0.390824	-1.35541	0.004841	down
A0A7G2EF89	(thale cress) hypothetical protein	0.421192	-1.24745	0.001073	down
A0A8F5Z876	Photosystem II CP47 reaction center protein	0.430064	-1.21738	0.172297	down
O80517	Uclacyanin-2	0.446814	-1.16225	0.003591	down
A0A178VRV6	Oleosin	0.459325	-1.12241	0.003028	down

Table S3. DEPs related to Arabidopsis root development

Accession	Protein names	Gene	FC	Log2FC	fdr	Regulated
Q96511	Peroxidase 69	AT5G64100	0.498771856	-1.003548033	0.001756581	Down
A0A178VGZ7	Peroxidase	AT3G49120	0.652516948	-0.615912721	0.008090193	Down
P25818	Aquaporin TIP1-1	AT2G36830	0.494870667	-1.014876564	0.007068383	Down

Table S4. 6 DEGs were identified in the phenylpropanoid biosynthesis pathway

Gene id	Gene names	BaseMean	FoldChange(FT/CT)	Log2FoldChange	Pval	Padj	Regulated
AT1G68850	Peroxidase superfamily protein	295.0966261	397.6953035	192.4979487	0.484033749	-1.046820453	down
AT2G39040	Peroxidase superfamily protein	51.08864544	76.02362933	26.15366154	0.344020165	-1.539434961	down
AT5G24070	Peroxidase superfamily protein	28.71511762	40.74532508	16.68491015	0.409492626	-1.288090623	down
AT5G64100	PRX69	4394.557964	6883.475376	1905.640551	0.276842793	-1.852861128	down
AT2G35380	Peroxidase superfamily protein	435.6569879	643.4423772	227.8715986	0.354144531	-1.497589834	down
AT5G17820	Peroxidase superfamily protein	1163.122854	1607.808057	718.4376515	0.446842923	-1.162160321	down
AT3G01190	Peroxidase superfamily protein	1151.579415	1544.674179	758.4846516	0.49103213	-1.026110668	down
AT5G64110	Peroxidase superfamily protein	142.8346653	215.0024098	70.66692083	0.328679669	-1.605245877	down
AT2G38390	Peroxidase superfamily protein	252.6615094	369.5270484	135.7959703	0.367485874	-1.444239301	down
AT3G03670	PER28	53.03814342	105.5762092	0.500077667	0.004736651	-7.721916877	down
AT1G67980	CCOAMT	459.1608376	692.162359	226.1593163	0.32674316	-1.61377106	down

Table S5. 6 DEPs were identified in the phenylpropanoid biosynthesis pathway

Gene Symbol	Protein names	FC	Log2FC	P.value	Regulated
PER69	Peroxidase 69	0.49877186	-1.003548	3.4977E-06	down
PER32	Peroxidase 32	0.53378378	-0.9056726	1.5011E-05	down
PER70	Peroxidase 70	0.5366702	-0.8978923	3.0331E-05	down
AXX17_At3g43260	Peroxidase	0.65251695	-0.6159127	0.00016267	down
AN1_LOCUS10302	Peroxidase	0.5089406	-0.9744308	0.00024218	down
AN1_LOCUS13615	Peroxidase	0.65180852	-0.6174799	0.00011746	down
AT5G17820	Peroxidase 57	0.63676381	-0.6511698	1.946E-05	down

Table S6. Tau family of glutathione-S-transferases (GSTs)

Gene id	Gene names	BaseMean	FoldChange(FT/CT)	Log2FoldChange	Pval	Padj	Regulated
AT1G17170	GSTU24	1218.457956	2.177719033	1.122817831	1.11426E-30	7.44929E-28	up
AT1G78360	GSTU21	8.933350785	0.170778834	-2.549798917	5.35752E-05	0.001243658	down
AT1G11540	Sulfite exporter TauE/SafE family protein	42.35933999	0.486506719	-1.039468365	0.000114159	0.00238713	down
AT2G29470	GSTU3	16.83119539	2.303712449	1.20396065	0.00389468	0.045047764	up
AT1G78370	GSTU20	194.6342985	2.02777792	1.019899659	0.013764602	0.118367482	up

Table S7. 6 DEGs were identified in the auxin pathway

Gene id	Gene names	BaseMean	FoldChange(FT/CT)	Log2FoldChange	Pval	Padj	Regulated
AT4G13790	SAUR25	39.16659606	25.16018257	53.17300955	2.113379321	1.079551733	up
AT2G21220	SAUR12	22.89508395	13.82952026	31.96064764	2.311045289	1.208545532	up
AT1G15050	Belongs to auxin inducible gene family	28.11730787	16.69703458	39.53758116	2.367940306	1.243632712	up
AT4G09530	SAUR17	22.71644085	13.61595762	31.81692408	2.33673789	1.224495917	up
AT1G48660	Auxin-responsive GH3 family protein	18.10639088	8.992327865	27.22045389	3.027075335	1.59792458	up
AT5G51470	Auxin-responsive GH3 family protein	22.16176997	31.38738338	12.93615655	0.412145109	-1.27877572	down
AT1G23160	GH3.7	35.34434888	54.33593848	16.35275928	0.3009566	-1.732372642	down

Table S8. Parameters settings of LC-MSMS raw data for protein database searching

Item	Parameters Settings
Type	Reporter ion MS2
Isobaric labels	TMT 10plex
Enzyme	Trypsin
Reporter mass tolerance	0.005 Da
Max Missed Cleavages	2
Main search Peptide Tolerance	10 ppm
MS/MS Tolerance	0.02 Da
Fixed modifications	Carbamidomethyl (C), TMT6plex (K), TMT6plex (Peptide N- term)
Variable modifications	Oxidation (M) , Acetyl (Protein N-term)
Database	uniprot-Arabidopsis thaliana[3702]-136433-20230330.fasta
Database pattern	Target-Reverse
PSM FDR	≤0.01
Protein FDR	≤0.01
Protein quantification	Razor and unique peptides were used for protein quantification.
